# The effect of physical activity on psychological distress, cortisol and obesity: results of the farming fit intervention program

**DOI:** 10.1186/1471-2458-13-1018

**Published:** 2013-10-28

**Authors:** Susan Brumby, Ananda Chandrasekara, Peter Kremer, Susan Torres, Scott McCoombe, Paul Lewandowski

**Affiliations:** 1 National Centre for Farmer Health, Western District Health Service, PO BOX 283 Hamilton, Vic 3300 Australia; 2 School of Medicine, Deakin University, Geelong, Vic 3216 Australia; 3 School of Exercise and Nutrition Sciences, Deakin University, Geelong, Vic 3216, Australia; 4 School of Exercise and Nutrition Sciences, Deakin University, Burwood, Vic 3125, Australia

**Keywords:** Obesity, Cortisol, Mental health, Rural, Farmer

## Abstract

**Background:**

Rural and regional Australians have a higher likelihood of mental illness throughout their lifetime than people living in major cities, although the underlying reasons are not yet well defined. Additionally, rural populations experience more lifestyle associated co-morbidities including obesity, diabetes and cardiovascular disease. Research conducted by the National Centre for Farmer Health between 2004 and 2009 revealed a positive correlation between obesity and psychological distress among the farming community. Chronic stress is known to overstimulate the regulation of the hypothalamic-pituitary-adrenal (HPA) axis and cortisol secretion which are associated with abdominal adiposity. Increasing physical activity may normalise cortisol secretion and thereby positively impact both physical and mental health. This paper assesses the effects of increasing physical activity on obesity, health behaviors and mental health in Victorian farming men and women.

**Methods:**

Farming Fit was a six month quasi-experimental (convenience sample) longitudinal design control-intervention study. Overweight or obese (BMI ≥25 kg/m^2^) farm men (*n* = 43) and women (*n* = 29) were recruited with demographic, health behaviors, anthropometric, blood pressure and biochemistry data collected at baseline and at a six months. Salivary cortisol and depression anxiety stress scale results were collected at baseline, three and six months. The intervention group (*n* = 37) received a personalized exercise program and regular phone coaching to promote physical activity.

**Results:**

The intervention group showed significant reductions in body weight and waist circumference. Results indicated that following the six month exercise program, the intervention group were 2.64 ± 0.65 kg lighter (*p* < 0.001), had reduced waist circumference by 2.01 ± 0.86 cm (*p* = 0.02) and BMI by 0.97 ± 0.22 kg/m^2^ (*p <* 0.001) relative to the control group.

**Conclusion:**

Increasing physical activity altered measures of obesity in farm men and women but did not affect mental health measures or cortisol secretion levels.

**Trial registration:**

ACTRN12610000827033

## Background

Mental health disorders are one of the largest contributors to disability-adjusted life years (DALYs) worldwide [1]. The 2007 National Survey of Mental Health and Wellbeing found that one in five (20%) Australian adults experience mental illness in any year [2]. Additionally, one in four of these adults experience more than one mental disorder [2]. In 2010, anxiety and depression were the second largest burden in terms of disability-adjusted life years (DALYs) in Australia [3] and intentional self-harm or suicide the 10th leading cause of all registered deaths in 2008 [4]. Recent studies indicate that rural and regional Australians have a higher likelihood of suffering a mental disorder throughout their lifetime than people living in major cities [3], although the reasons underlying this imbalance remain poorly defined.

Obesity is also an increasing issue worldwide [5]. In 2007–08, the Australian Bureau of Statistics (2009) National Health Survey determined that approximately 62% of adults aged 18 and over were overweight or obese [6]. Rural populations experience poor outcomes related to mental illness and the lifestyle associated co-morbidities of obesity, diabetes and cardiovascular disease [7,8]. Research conducted by the National Centre for Farmer Health between 2004 and 2009 revealed a positive correlation between obesity and psychological distress among farming men and women [9,10]. These physical and mental health co-morbidities are exacerbated by environmental constraints including drought, flood and bushfires, access to rural healthcare, geographic and social isolation, costs of health service access and the distance-decay effect [11].

Previous studies on chronic psychological distress [12] and our own preliminary cross sectional data analysis [9] have established links between physical activity and mental wellbeing. In the Farming Fit study we hypothesized that prolonged levels of stress experienced by farm men and women increased the levels of the hormone cortisol and left them vulnerable to the defeat response [9,13] subsequently impacting on physical and mental health. Chronic exposure to circulatory cortisol can adversely impact fat storage, resulting in abdominal adiposity, weight gain and, in turn, further cortisol release [13]. As reported by Schwarz *et al.*[14], improving physical activity decreases fat storage and increases endorphin concentrations via the hypothalamic-pituitary-adrenal (HPA) axis. Physical activity has the added benefits of also improving mood state and reducing circulating cortisol levels [14]. Drawing on these findings, the Farming Fit study [9] was undertaken to explore the interrelationships of these factors among a selected group of farming men and women in Victoria, Australia [10].

## Methods

### Participants

Consenting overweight or obese (as determined by body mass index (BMI) ≥ 25 kg/m^2^) farm men and women were enrolled into the 6-month quasi-experimental control-intervention study. Participants were participating in the Sustainable Farm Families (SFF) programs [15] where each program consisted of 15–20 farm men and women. SFF programs were pre-assigned to either control or intervention groups with participants from a given program being assigned to the same group, thereby minimizing the potential for any contamination effects or selection biases. This pre-assignment of study groups to control or intervention based on SFF program location, resulted in a limitation of the study, that being individual participants were not completely randomized. All participants had been farming for more than 5 years, were aged between 18 and 75 years, spoke English and lived 10 kilometers or more from a regional center with a population greater than 10,000 people. Participants were excluded from the study if they had a chronic illness, were pregnant, lactating or unable to participate in the physical activity program. Participants whom fulfilled the abovementioned criteria were pre-assigned to intervention or control groups.

### Design

Participants allocated to the intervention group were provided with;

1. An individualised exercise program designed by an exercise physiologist and undertaken over the 6-month period.

2. Access to an exercise physiologist and research assistant to provide consultation on individualised exercise programs and coaching over the 6-month period.

3. Regular monitoring of exercise activity and physical goal attainment by phone, email or text message at least fortnightly.

### Measures

Both groups undertook health assessments in which baseline data was collected on anthropometric parameters, percentage body fat and blood pressure. Baseline biochemical analysis of venous blood was also performed to attain glucose, plasma cortisol, plasma total cholesterol, plasma triglycerides, plasma high-density lipoprotein (HDL) and plasma low density lipoprotein (LDL) levels following a 10 hour fast. Commensurate anthropometric and biochemical analyses were performed at three and six-month intervals as detailed in Tables 1 and 2.

**Table 1:** Summary characteristics at baseline and follow-up for intervention and control groups and all participants

	**Intervention**		**Control**		**Total**	
**Measure**	**Baseline**	**Follow-up (6mths)**	**Baseline**	**Follow-up (6mths)**	**Baseline**	**Follow-up (6mths)**
	**(n = 35)**	**(n = 34)**	**(n = 37)**	**(n = 34)**	**(n = 72)**	**(n = 68)**
Gender (female,%)	42.9		37.8		40.3	
Age (years)	50.14(11.64)		53.22(9.32)		51.72(10.55)	
Height (cm)	171.79(9.70)		172.01(7.65)		171.90(8.64)	
Weight (kg)	94.99(14.62)	92.83(14.03)	90.48(12.83)	91.03(12.39)	92.67(13.82)	91.93(13.17)
BMI (kg/m^2^)	32.12(3.37)	31.51(3.32)	30.55(3.65)	30.86(3.90)	31.31(3.58)	31.19(3.61)
Body fat (%)	35.30(7.25)	34.81(7.31)	33.43(7.47)	33.21(9.65)	34.34(7.37)	34.01(8.54)
Waist circumference (cm)	102.74(10.48)	101.12(9.86)	101.80(10.42)	102.35(10.34)	102.26(10.38)	101.74(10.47)
Hip circumference (cm)	111.74(7.99)	110.29(7.99)	108.05(7.76)	108.85(7.26)	109.85(8.03)	109.57(7.61)
Waist to hip ratio	0.92(0.08)	0.92(0.07)	0.94(0.08)	0.94(0.08)	0.93(0.08)	0.93(0.08)
DASS total score	8.26(7.91)	8.24(6.20)	7.11(5.74)	6.00(4.55)	7.68(6.87)	7.10(5.50)
Fasting blood glucose (mmol/L)	5.21(0.94)	5.14(0.68)	5.02(0.73)	5.19(0.69)	5.11(0.83)	5.16(0.68)
Total cholesterol (mmol/L)	5.62(1.02)	5.37(0.89)	5.93(0.98)	5.61(0.95)	5.78(1.01)	5.49(0.92)
Triglyceride (mmol/L)	1.49(0.50)	1.27(0.54)	1.63(0.64)	1.49(0.66)	1.56(0.58)	1.32(0.61)
HDL cholesterol (mmol/L)	1.46(0.34)	1.42(0.32)	1.48(0.37)	1.42(0.31)	1.47(0.35)	1.42(0.31)
LDL cholesterol (mmol/L)	3.47(0.97)	3.36(0.80)	3.71(0.89)	3.50(0.82)	3.60(0.93)	3.43(0.81)
Blood cortisol (nmol/L)	417.80(128.90)	373.68(131.12)	373.86(109.47)	377.68(120.67)	395.22(120.51)	375.68(125.07)
Systolic blood pressure (mmHg)	132.61(17.30)	128.03(12.84)	136.45(15.74)	128.01(13.65)	134.59(16.51)	128.02(13.15)
Diastolic blood pressure (mmHg)	86.29(8.78)	82.44(7.61)	87.03(9.71)	81.57(8.90)	86.67(9.21)	82.00(8.23)
Pulse rate (beats/minute)	70.4(9.59)	67.44(8.64)	69.65(8.13)	66.94(7.33)	70.01(8.81)	67.19(7.97)

Data expressed as mean±SD or proportions. Intervention and control groups were not different at baseline for all measures.

**Table 2:** Adjusted differences in outcome measures between intervention and control groups at follow-up for total sample

**Measure**	**Difference**	**SE**	***P***	**95% CI**
Weight (kg)	**-2.64**	**0.65**	**<0.001**	**-3.93, -1.35**
Body mass index (kg/m^2^)	**-0.97**	**0.22**	**<0.001**	**-1.42, -0.53**
Waist circumference (cm)	**-2.01**	**0.86**	**0.02**	**-3.73, -0.29**
Waist to hip ratio	-0.01	0.01	0.67	-0.02, 0.01
Body fat percentage	-0.22	1.14	0.85	-2.50, 2.06
DASS total score	1.26	1.16	0.28	-1.06, 3.58
Fasting blood glucose (mmol/L)	-0.13	0.15	0.36	-0.43, 0.16
Total cholesterol (mmol/L)	-0.01	0.19	0.95	-0.39, 0.37
Triglyceride (mmol/L)	-0.15	0.12	0.23	-0.40, 0.10
HDL cholesterol (mmol/L)	0.00	0.04	0.97	-0.08, 0.08
LDL cholesterol (mmol/L)	0.03	0.18	0.86	-0.32, 0.39
Blood pressure (systolic) (mmHg)	2.20	2.30	0.34	-2.39, 6.81
Blood pressure (diastolic) (mmHg)	1.24	1.69	0.47	-2.14, 4.61
Pulse rate (pulse/minute)	0.21	1.80	0.91	-3.39, 3.80
Blood cortisol (nmol/L)	-16.13	29.90	0.59	-75.86, 43.59

Adjusted for baseline measure; Results significantly different (*p* < 0.05) are bolded.

The Depression and Anxiety Stress Scale (DASS21) [16] psychological questionnaire was completed at baseline, three months and at completion of the project at six months. People with very high levels of psychological distress (DASS 21 Score depression >28 or anxiety >20 or stress >37) [17] were referred for mental health assessment by a qualified health professional. Participants also reported physical activity output and provided saliva samples for cortisol analysis at baseline, three month and six month follow up. Saliva samples were provided four times during the day (9 am, 12 noon, 4 pm and 8 pm) and collected by a salivette sampling device (Sarstedt). Subjects were requested to abstain from smoking; ingesting caffeine, alcohol, food and all fluids; and strenuous physical activity for 1 hour before each saliva collection. All saliva samples were returned to the laboratory for analysis within 5 days of collection via mail. On arrival at the laboratory saliva was collected from the salivette sampling device by centrifuging at 3000 ×*g* for 5 min, at room temperature, and then stored at -80 C until saliva cortisol levels are assayed by radioimmunoassay (Orion Spectra Cortisol®).

### Statement of ethics

Ethics approval was obtained from Deakin University Human Research Ethics Committee (HREC 2009/215 dated 03/02/2010) which conforms to the provisions of the Declaration of Helsinki in 1995 (as revised in Tokyo 2004).

### Data analysis

Data are expressed as mean ± (SD), unless otherwise stated. Data analyses were performed on all anthropometric, mental health, metabolic/biochemical, and physical activity measures. Differences in baseline anthropometry, mental health, biochemical, physical activity and personal variables were determined by separate t-tests, Chi-square tests and ANOVA (total salivary cortisol). Differences in follow-up anthropometry, mental health, biochemical (except salivary cortisol) measures were determined by separate generalized linear models (GLM) with group (intervention or comparison) entered as the predictor along with the baseline measure. Differences for salivary cortisol were assessed using a 3-way ANOVA test with test period and time as ‘within subject variables’ and group as a ‘between groups variable’. Checks on the self-reported physical activity (PA) data revealed a number of outlier scores. Consequently, any outlier scores (> ±3 SD from mean) on each of the three PA variables were removed. This influenced a total of four cases of PA across the three time points. Differences for PA were assessed using a 2-way ANOVA test with test period as a ‘within subject variable’ and group as a ‘between groups variable’. Where ANOVA revealed significant interactions these effects were further assessed using separate t-tests. ANOVA results were interpreted using *df* corrected (Greenhouse-Geisser) values as appropriate [18]. All analyses were conducted using SPSS v20 and statistical significance set at *p* < 0.05.

## Results

### Characteristics of sample

The Farming Fit intervention was applied over a period of six months. Retention rates for both groups were very high (intervention: 97%; control: 92%), with one participant from the intervention group and three from the control group unavailable for follow-up due to other commitments and climatic events. Table 1 shows the characteristics of the intervention, control and combined groups at baseline and 6 month follow-up. At baseline, the total participants were overweight or obese (BMI 31.3 ± 3.58), ranged in age from 33 – 72 years with an average age of 51.7 ± 10.6 years, and were predominantly male (59.7%). Average baseline DASS score of the intervention group was 8.26 ± 7.91 and for the control group was 7.11 ± 5.74. Results of *t-*test and Chi-square analyses for differences among the intervention and control groups at baseline were all non-significant, indicating equivalence.

Total salivary cortisol profiles across the four time points for baseline, and subsequent follow-up periods are summarized in Table 3. Results of ANOVA indicated that at baseline, there was a significant change in salivary cortisol levels over the course of a day, with a peak at 9 am that was significantly higher than the 4 pm and 8 pm time points (*Fcorr*1.4, 85 = 94.7, *p* < .001), but no effect for group or the group x time interaction (*F*’s < 2.0, *p*’s > .05). Similar results of diurnal variation, with a peak at the start of the day (9 am) followed by a decline over the course of the day have been reported elsewhere [19]. However, the variability of salivary cortisol between individuals and the modest cohort size did not allow any significant differences to be detected between baseline and 6 month follow-up. In addition, the two-way and three-way interactions were all non-significant indicating that salivary cortisol profiles across test, time and the combination of test x time were not significantly different for either control or intervention groups. This change in salivary cortisol levels over the course of the day is expected as cortisol follows a diurnal pattern with peak levels following awakening and declining levels thereafter [19].

**Table 3:** Total salivary cortisol (nmol/L) for intervention and control groups at baseline, 3 months and follow-up

	**Baseline**				**3 months**				**Follow-up**			
	**9 am**	**12 pm**	**4 pm**	**8 pm**	**9 am**	**12 pm**	**4 pm**	**8 pm**	**9 am**	**12 pm**	**4 pm**	**8 pm**
Intervention (n = 35)	23.1(17.3)	11.8(7.3)	7.3(4.6)	4.7(3.2)	24.8(16.9)	12.0(6.8)	7.4(4.1)	4.3(2.8)	31.3(31.0)	17.2(26.3)	11.7(23.2)	7.0(13.9)
Control (n = 37)	24.5(13.2)	14.7(6.5)	8.4(4.7)	3.8(2.5)	32.2(30.3)	19.2(26.5)	13.5(24.0)	7.8(14.9)	26.3(18.0)	14.6(12.1)	9.6(11.0)	5.4(7.9)
Total (n = 72)	25.4(15.4)	13.3(7.0)	7.9(4.6)	4.2(2.9)	28.7(25.0)	15.8(20.1)	10.6(17.8)	6.2(11.1)	28.7(25.0)	15.8(20.1)	10.6(17.8)	6.2(11.1)

Data expressed as mean±SD, n varies across test periods.

### Anthropometry and mental health outcome measures

Results of analyses by control and intervention group for the anthropometric and mental health outcome measures are presented in Table 2. These analyses revealed significant improvements on key anthropometric measures for the intervention group relative to the control group. Specifically, at 6 month follow-up, the intervention group were 2.64 ± 0.65 kg lighter (*p* < 0.001), had a 2.01 ± 0.86 cm lower waist circumference (*p* = 0.02) and 0.97 ± 0.22 kg/m^2^ lower BMI (*p* < 0.001) relative to the control group. There were no significant differences for waist-to-hip ratio or body fat percentage. There was also no difference between groups for overall mental health (DASS total score) at follow up.

### Metabolic and biochemical measures

Results of group metabolic and biochemical measures are also presented in Table 2. No significant group effect was found for systolic and diastolic blood pressure, pulse rate measures, blood glucose, blood total cholesterol, triglycerides, HDL, LDL and blood cortisol.

### Physical activity

Results of physical activity data (trimmed) as self-reported on the International Physical Activity Questionnaire (IPAQ) [20,21] are presented in Table 4. The data revealed no significant difference in physical activity levels at baseline between the control and intervention groups (*t* < 2.0). Results of ANOVA revealed no significant effect for either group or test period (*F*’s < 2.0). There was however a significant group x test period interaction (*F*(2,120) = 6.0, *p* < .01) indicating significant group differences for changes in physical activity across the three time periods. Follow-up analyses revealed no differences for physical activity at 3 months (*t* < 2.0), but at 6 months the intervention group were significantly more physically active than the control group (*t*(31) = 2.8, *p* < .01). This result is indicated by the respective changes in physical activity levels from baseline to 6 month follow-up for the two groups: the intervention group had a (94.4%) increase in physical activity (minutes/week) when compared with the control group (18.9% reduction) as shown in Table 4.

**Table 4:** Mean trimmed physical activity (minutes/week) for intervention and control and groups at baseline, 3 months and 6 month follow-up

**Group**	**Measurement**	**Mean (Std. Error)**	**95% Confidence interval**
Control	Baseline	992.26(135.36)	721.49-1263.03
	3 Month	962.58(131.91)	698.71-1226.45
	Final	804.35(141.73)	520.86-1087.85
Intervention	Baseline	700.32(135.36)	429.55-971.09
	3 Month	940.50(131.91)	676.63-1204.37
	Final	1361.26(141.73)	1077.76-1644.76

## Discussion

The purpose of the Farming Fit study was to examine the effect of physical activity on mental and physical health parameters in a group of farm men and women in Victoria, Australia. Participants of the intervention group displayed a significant reduction in measures of obesity (BMI and waist circumference). However, no effect on cortisol levels or depression, stress and anxiety were observed.

Furthermore, the study aimed to increase the level of daily physical activity within an intervention group at high risk of mental and physical health burden. An increase in physical activity was achieved in the intervention group via individualised exercise coaching programs together with regular monitoring of exercise and support provided. The level of daily physical activity reported by participants from the intervention and control group at base line was greater than the 210 minutes per week of moderate-intensity activity recommended in the Physical Activity Guidelines for Australians [22]. The level of physical activity recorded in the current study is in contrast to a number of previous investigations that suggest that people living in rural areas have low levels of physical activity [23] although these previous measurements were made comparing differences between urban and rural population activity levels [24,25]. This amount of self-reported moderate-intensity exercise within an overweight or obese farming cohort at baseline was likely skewed by reporting physical labour in addition to any structured exercise.

With participants in the intervention group undertaking an individualised exercise coaching program we identified an increase in physical activity equivalent to carrying out vigorous exercise on 3–4 days a week for 20 minutes or more each time. An increase in activity of that nature would see the participants of the intervention group satisfying the revised 2005 Physical Activity Guidelines for Australians. Previous research has suggested that the physical activity of people living in rural areas could be increased through the use of unstructured or low-intensity activity such as walking and tai chi as those activities do not require specialist facilities which are sparsely located in rural areas [23]. Irrespective of the methods used there is strong evidence linking increased physical activity as a method to prevent various chronic conditions, such as coronary heart disease, stroke, type 2 diabetes, cancer, obesity [26].

The study had a very high completion rate (94%) in comparison to other studies [27] with only four participants withdrawing from the project during its completion term (34 interventions, 34 controls) due to climatic conditions and time constraints. Participants also identified that they did not know how to exercise on the farm, lived too far away from gyms and/or found them too expensive with the cost of subscription, travel, fuel and time off farm. With the help of Farming Fit participants and the exercise physiologist, a DVD – called Farming Fit was produced post study. The DVD was designed to show farm men and women how best to utilise the infrastructure on their farm to assist them in strength training and stretches using workshop benches, doorways and steps. Over 2000 copies of the Farming Fit DVD have now been distributed free of charge to both farmers and organisations in rural areas [28]. However, there were several limitations to this project that need to be highlighted.

### Limitations

Due to time constraints, the geographical dispersion of SFF program locations, the Farming Fit program did not fully randomise the farmers to the intervention and control groups. Instead SFF programs were pre-assigned to control and intervention groups, with programs run earlier in the year assigned to the intervention group. Only 72 participants (35 interventions, 37 controls) of the anticipated 100 participants were able to be recruited from SFF programs due to a limited number of participants meeting the Farming Fit selection criteria. Therefore, this study was underpowered and may explain the lack of change with regards to the DASS and cortisol.

### Droughts and floods

The Farming Fit program commenced in early 2010 and included districts affected by the prolonged drought of 2004–2010. In January 2011, Victoria experienced some of the worst floods on record with some areas recording three to four times the January average rainfall [29]. Six of the Farming Fit programs occurred in areas where the flood impacted heavily on participant’s home and farming livelihood. Conversely in the other five programs the rain heralded the end of Victoria’s prolonged drought. It is impossible to estimate the effect of these unprecedented climatic conditions on the outcomes in the intervention and control groups particularly in relation to mental health and cortisol secretion.

## Conclusions

The Farming Fit study was designed to identify an effective method of counteracting psychological distress and obesity of farm men and women. This study demonstrates that individualised exercise coaching programs can also increase physical activity in farming populations, thereby conveying the associated health benefits to a demographic in need of improved non-communicable disease management and outcomes. It is noted that unprecedented climatic challenges are likely to have impacted on this study. However, understanding how physical health is important for the wellbeing of farmers and their enterprise may help to break the ‘defeat’ cycle during long periods of stress and assist the agricultural industry in addressing health issues in an accessible mode. Actions of further research and intervention among larger populations, over longer time periods and in conjunction with dietary modification are urgently required.

## Competing interests

The authors declare that they have no competing interests.

## Authors’ contributions

SB drafted the initial manuscript, undertook health assessments and oversaw data collection, AC undertook data analysis, undertook specific health assessments, supervised data collection and assisted with the initial manuscript, PK and PL undertook data analysis, interpretation and drafting of the manuscript. ST and SM assisted with drafting the manuscript. All authors contributed to the methods and design of the study and read and approved the final manuscript.

## Pre-publication history

The pre-publication history for this paper can be accessed here:

http://www.biomedcentral.com/1471-2458/13/1018/prepub
